# Supplementary material for: Performing Qualitative Mask Fit Testing Without a Commercial Kit: Fit Testing Which Can Be Performed at Home and at Work
Source: Disaster Med Public Health Prep. 2020 Sep 10:1–5. doi: 10.1017/dmp.2020.352 (PMC7642972; doi:10.1017/dmp.2020.352)
Supplement: Supplementary file 1 [file S1935789320003523sup001.pdf]

Data Supplement

The following pages include supplemental information to the article “Performing Qualitative Mask Fit Testing without a Commercial Kit: Fit Testing Which can be Performed at Home and at Work”.

There are three sections of supplementary information.

Observations & Data, pages 2 - 17

Photographs, pages 18 - 23

Preliminary Risk Analysis, pages 24 - 26

Authors for this information are:

Eugenia O’Kelly, Cambridge University
Anmol Arora, Cambridge University
Charlotte Pearson, University of Oregon
James Ward, Cambridge University
John Clarkson, Cambridge University.

Data Supplement - Data & Observations

Introduction

The following pages detail the qualitative and quantitative feedback from three testers evaluating alternative fit testing devices and enclosures.

Quantitative fit testing was used to evaluate the fit of the N95 and KN95 masks on the testers and thus provide a key to the correct answers for each mask. Quantitative tests were conducted using a TSI PortaCount Pro Respirator Fit Tester model 8038+, with N95 mask fit testing feature enabled following OSHA protocol 29CFR1910.134. The seven activities described by OSHA were undertaken by testers and a total 'fit factor' calculated from these seven activity scores. According to OSHA standards, an N95 mask must achieve a fit factor of at least 100 to be considered protective. The highest score possible for an N95 respirator is over 200, which is represented as 200+. The lower the score, the less well the mask fits the user. Masks with low fit factors should allow the user to taste the solutions used in qualitative fit tests. Quantitative fit factors for each tester are shown at the top of the data sheets for reference.

Time to taste sensation were recorded along with any notes about the position the testers used and their experience during testing.

Three testers took part in assessing the devices. Their notes are color coded for ease of reference. Tester 1 was the exploratory tester who evaluated all combinations of equipment. Their main job was to assess which pieces of equipment showed promise and which had insurmountable problems, as well as to assess the time it took for each device to provide a taste sensation. Tester 1 completed a full set of tests on each device. Data from Tester 1 is Blue. The job of Tester 2 was to provide an independent analysis of each device and enclosure. Tester 2 trialed each device in a limited number of test modes. They completed full tests on promising devices. Data from Tester 2 is Green. Tester 1 and Tester 2 conferred after testing to compare results and discuss the best options for replacement devices and enclosures. If any discrepancies were found between Tester 1 and Tester 2's experiences, further testing would conducted. This was not the case, as the findings of Tester 1 and Tester 2 closely agreed. Tester 3 provided further evaluation of the most promising devices/enclosure combinations. Data from Tester 3 is Pink.

Tester 1 - Exploratory Tester

The purpose of the exploratory tester was to trial initial devices and check what devices are promising and worth further study.

Tester #1 - Quantitative Fit Scores

N95 Mask	200+ (pass)
KN95 Mask	1.9 (fail)

No Testing Enclosure

Aroma Diffuser	Placement Notes	Taste / No Taste	Time to Taste	Notes
Sensitivity Test	Sitting a foot away from . Head slightly above machine where the air is coming out.	Taste	10 Sec	Tasted the solution quickly and very strongly.
KN95		Taste	14 Sec	I think I tasted the solution before the 14 seconds, but it was so subtle that I waited to be sure
N95		No Taste	60 sec	Not even a hint of sweetness was detected.
Nose Gap N95		Taste	41 Sec	Tasted the solution, but not a strong taste
Chin Gap N95		Taste	51 Sec	Tasted the solution, but not a strong taste
Cheek N95		No Taste	60 sec	About halfway through there was a slight hint of sweetness, but I waited one more breath to see if it got sweeter. It did not so I kept going.

Humidifier	Placement Notes	Taste the Solution?	Time to Taste	Notes
Sensitivity Test	Wand set up on table. I started a few feet away and moved closer till I was directly over the vent.	No Taste	1 hour	I did not taste the solution. I could sometimes taste it on my lips when I licked them, but was completely unable to taste it in my mouth.

Ultrasonic Mist Maker	Placement Notes	Taste the Solution?	Time to Taste	Notes
Sensitivity Test	Mist maker in container 3-4 inches away from face	Taste	12 Sec	Sweet taste
KN95		No Taste	1 Min	The Cloud of vapor was dense, but wet
N95		No Taste	1 Min	No taste in the slightest. At the distance I am at the mask only gets slightly wet.
Nose Gap N95		Taste	52 Sec	Some taste at the end of the test.
Chin Gap N95		No Taste	1 Min	At one point I thought I tasted the sweet solution, but it was too light.
Side of Face Gap N95		No Taste	1 Min	No taste. The water kept spraying me in the face.

Spray Bottle	Placement Notes	Taste the Solution?	Time to Taste	Notes
Sensitivity Test	I am holding the bottle a foot away, spraying towards my face.	Taste	1 Spray	Very sweet right away.
KN95		Taste	1 Spray	Could not taste as clearly with the mask.
N95		No Taste	10 Sprays	Could not taste anything.
Nose Gap N95		Taste	1 Spray	Could taste it slightly.
Chin Gap N95		No Taste	10 Sprays	I thought I tasted it at one point but it faded quickly.

Side of Face Gap N95		No Taste	10 Sprays	I thought I tasted it at one point but it faded quickly.

Hood

Aroma Diffuser	Placement Notes	Taste the Solution?	Time to Taste	Notes
Sensitivity Test	Diffuser held directly under curved pipe	Taste	3 Sec	This hood makes testing go more quickly
KN95		Taste	5 Sec	The top of the diffuser is tilted. I noticed the fog came quicker if the top is tilter towards the mask.
N95	The fog truly surrounded me by now	No Taste	60 sec	I could tell a slight amount of the solution was saturating the air of the mask
Nose Gap N95		Taste	5 Sec	The fog is so dense in the mask that the fog can find any hold in the mask quickly
Chin Gap N95		Taste	10 Sec	With the hood, the fog is able to surround the mask entirely. Without the hood the fog hits a few places on the mask and then disperses.
Side of Face Gap N95		Taste	15 Sec	

Humidifier	Placement Notes	Taste the Solution?	Time to Taste	Notes
Sensitivity Test	Opening of humidifier directly under curved pipe	No Taste	60 seconds	

Ultrasonic Mist Maker	Placement Notes	Taste the Solution?	Time to Taste	Notes

Sensitivity Test		Taste	2 Sec	Made me cough.
KN95		Taste	4 Sec	The water magically turns through the pipe and is able to hit you in the face, making your mask wet.
N95		No Taste	60 sec	When I breathed in I pulled the fog into the mask.
Nose Gap N95		Taste	4 Sec	
Chin Gap N95		No Taste	1 Min	The pipe is getting very wet.
Side of Face Gap N95		Taste	15 Sec	The taste came gradually .

**Spray Bottle**	**Design**	**Taste the Solution?**	**Time to Taste**	**Notes**
Sensitivity Test	I sprayed the solution straight into the hole of the hood. No pipe used.	Taste	1 Spray	Sprayed the device from the hole which is about an inch away from my mouth.
KN95		No Taste	10 sprays	Mask got fairly wet.
N95		No Taste	10 Sprays	Mask got fairly wet.
Nose Gap N95		Taste	8 Sprays	Weak taste - mask is getting quite wet.
Chin Gap N95		Taste	6 Sprays	Weak taste - I could clearly taste the solution but it was very weak
Side of Face Gap N95		No Taste	10 sprays	Mask is very wet now

Bag

Aroma Diffuser	Design	Taste the Solution?	Time to Taste	Notes
Sensitivity Test	Head in bag - hard to get bag down far enough to concentrate smoke around chin and bottom of mask area	Taste	4 s	Performed sensitivity test with full-strength solution. I think, for those looking to perform these tests at home, they might not need to have an extra sensitivity solution.
KN95	Head in bag	Taste	4s	
N95		No Taste	60 sec	
Nose Gap N95		taste	5 s	
Chin Gap N95		Taste	27 s	Can be hard to get mist to reach bottom of mask if head is tilted forward and bag does not reach down to chin. A wider bag would be better.
Side of Face Gap N95		Taste	4 s	

Humidifier	Design	Taste the Solution?	Time to Taste	Notes
Sensitivity Test	Opening of humidifier into the bottom of bag	No Taste	60 seconds	

Ultrasonic Mist Maker	Design	Taste the Solution?	Time to Taste	Notes
Sensitivity Test	Allergies on day - test run by tester 2			

Spray Bottle	Design	Taste the Solution?	Time to Taste	Notes
Sensitivity Test	Could not fit spray bottle in bag			

Box

Aroma Diffuser	Design	Taste the Solution?	Time to Taste	Notes
Sensitivity Test	Head and diffuser at same level instead of head above diffuser.	Taste	30 Sec.	It took so long to taste that eventually I moved my hear upward and then I was able to taste it. The trick with the diffuser is being above the lip of the device.
KN95	Head above the diffuser from beginning.	Taste	15 Sec	With the head about 2 in. up and away from the device, it makes the taste come much quicker.
N95	Head above the edge of box - inside the box both the box and the diffuser are being held up.	No Taste	60 sec	Box doesn't seem to be airtight - the mist is not concentrating.
Nose Gap N95		Taste	10 Sec	Hard to create gap and hold box and testing device
Chin Gap N95		Taste	35 Sec	Taste was about half of what it usually is, but it was still prevalent.
Side of Face Gap N95		Taste	55 Sec	I could tell that each breath was sweet.

Humidifier	Design	Taste the Solution?	Time to Taste	Notes
Sensitivity Test	I put the humidifier under the box for one hour. I went under and tested for 1 minute. I did this twice to see if it was working but it was clear neither were successful.	No Taste	1 Hour	

**Ultrasonic Mist Maker**	**Design**	**Taste the Solution?**	**Time to Taste**	**Notes**
Sensitivity Test	Head in a box with the mist maker held below chin	Taste	4 Sec	Very wet
KN95		No taste	60 sec	The fog was not able to rise high enough to reach my face within the box
N95		No Taste	60 sec	Box does not help in the least
Nose Gap N95		Taste	7 Sec	Placed the nose gap right in front of the mist maker.
Chin Gap N95		Taste	45 Sec	Once again put the gap very close to the smoke. Only received a weak taste.
Side of Face Gap N95		No Taste	60 sec	The box made me get closer that I would have initially, but other than forcing proximity , I don't think the box helped much. Everything is wet.

**Spray Bottle**	**Design**	**Taste the Solution?**	**Time to Taste**	**Notes**
Sensitivity Test	Wearing box and mask spraying from a few inches away	Taste	1 Spray	Taste is strong. The box might be best here just for keeping things clean from the spray .
KN95		Taste	8 Sprays	Weak taste & mask is quite wet after one test.
N95		No Taste	10 Sprays	Getting wet and sticky!
Nose Gap N95		Taste	6 Sprays	I was uncertain at 4 sprays but at 6 I was certain.
Chin Gap N95		Taste	10 Sprays	At about 6-8 sprays I though I tasted something but it was subtle.

Side of Face Gap N95		Taste	10 Sprays	At about 6-8 sprays I though I tasted something but it was subtle.

Tester 2 - Confirmation Tester

The second tester trialed each device and enclosure in at least one combination to provide an independent analysis. Tester 1 and Tester 2 discussed the pros and cons of each piece of equipment, deciding what equipment had significant problems and what equipment warranted further testing.. Tester 2 completed full tests on the promising test devices/enclosures.

Tester #2 - Quantitative Fit Scores

N95 Mask	200+ (pass)
KN95 Mask	2.5 (fail)

No Testing Enclosure

Humidifier	Design	Taste the Solution?	Time to Taste	Notes
Sensitivity Test	Face held over opening of humidifier	No Taste	60 sec	I cannot taste anything, but maybe the vapor is a bit sticky?

Ultrasonic Mist Maker	Design	Taste the Solution?	Time to Taste	Notes
Sensitivity Test	Face above and in mist, which is quite wet	Taste	16 sec	This makes everything wet - a problem for mask testing

Aroma Diffuser	Design	Taste the Solution?	Time to Taste	Notes

Sensitivity Test	Head above mist generation	Taste		This is very promising. I can see one would either need to move this device around or concentrate it with an enclosure to ensure the mist reaches all edges of the mask

**Spray Bottle**	**Design**	**Taste the Solution?**	**Time to Taste**	**Notes**
Sensitivity Test	Spraying a little less than arms reach away	Taste	1 spray	Sweet as it hit my tongue, but the 'heaviness' of these drops might have trouble when gaps are not in line with the mouth.

NOTE: After discussion between tester 1 and 2, the spray bottle was disqualified over concerns about the size of the droplets produced being too large for effective mask gap testing.

Box

**Humidifier**	**Design**	**Taste the Solution?**	**Time to Taste**	**Notes**
Sensitivity Test	Face held over opening of humidifier	No Taste	60 sec	No taste at all

**Aroma Diffuser**	**Design**	**Taste the Solution?**	**Time to Taste**	**Notes**

Sensitivity Test		Taste		This did not concentrate the mist. I had to move close to the device - negating the benefit of the box. I could see the mist flowing in the opposite direct to my face and towards a corner of the box, where it seemed to escape in a fabric seam.

NOTE: Ultrasonic mist maker not tested, as it was difficult to get mist to flow into hood. Amount of mist flowing into the hood at any one time was unpredictable.

Hood

Humidifier	Design	Taste the Solution?	Time to Taste	Notes
Sensitivity Test	Face held over opening of humidifier	No Taste	60 sec	

Aroma Diffuser	Design	Taste the Solution?	Time to Taste	Notes
Sensitivity Test	Diffuser held directly under curved pipe	Taste		
KN95		Taste		
N95		No Taste		
Nose Gap N95		Taste		

Chin Gap N95		Taste		
Side of Face Gap N95		Taste		

Aroma Diffuser	Design	Taste the Solution?	Time to Taste	Notes
Sensitivity Test	Head in bag, top of aroma diffuser discharging into bag about 1" in front of face. My head fits into the bag better than Tester 1's head.	Taste	2 sec	Quick, strong, and clear taste
KN95		Taste	2 sec	Fast, easy to detect
N95		No Taste	60 sec	Can see fog building in bag and wrapping around mask
Nose Gap N95		Taste	2 sec	
Chin Gap N95	Lowered aroma diffuser spout a half inch to ensure fog traveled around chin	Taste	7 sec	
Side of Face Gap N95		Taste	3 sec	

NOTE: Full test completed to evaluate promising device/enclosure

Ultrasonic Mist Maker	Design	Taste the Solution?	Time to Taste	Notes
Sensitivity Test	Mist maker in solution container held at opening of bag	Taste	3 sec	Face gets wet, direct inhalations of mist causes coughing

Bag

KN95		Taste	60 sec	Large gaps cause coughing
N95		Taste	6 sec	Mask is very wet. Face is sticky.
Nose Gap N95		Taste	6 sec	
Chin Gap N95		Taste	10 sec	
Side of Face Gap N95		Taste	7 sec	

NOTE: Ultrasonic mist maker tested by Tester 2 due to allergies causing nasal congestion in Tester 1 on test day.
NOTE: Ultrasonic mist maker disqualified after discussion with Tester 1 due to its tendency to make the mask very wet.

**Humidifier**	**Design**	**Taste the Solution?**	**Time to Taste**	**Notes**
Sensitivity Test	Head held over opening of humidifier	No Taste	60 sec	

NOTE: Humidifier was disqualified after discussion with Tester 1 as it seems no taste is produced, no matter the configuration.

Tester 3 - Evaluating Successful Designs

The purpose of the third tester was to provide additional data on those device/enclosure combinations determined by testers 1 and 2 to be most promising. Note that this tester had an imperfect fit with the N95 mask. Thus, the tester should taste the test solution when wearing the N95 mask. The fit issue on this tester was subtle and due to a small gap under the chin. Thus only three tests could take place - a sensitivity test (correct value: taste), a KN95 test (correct value: taste), and a chin gap N95 (correct value: taste).

Tester #3 - Quantitative Fit Scores

N95 Mask	4.8 (fail)
KN95 Mask	1.3 (fail)

Hood

**Aroma Diffuser**	**Design**	**Taste the Solution?**	**Time to Taste**	**Notes**
Sensitivity Test	Diffuser held directly under curved pipe	Taste	4-6 sec	
KN95		Taste		
Chin Gap N95		No Taste	60 seconds	The hood seemed to block / prevent the mist from traveling beneath the mask to the chin area

Bag

**Aroma Diffuser**	**Design**	**Taste the Solution?**	**Time to Taste**	**Notes**
Sensitivity Test	Head in bag, top of aroma diffuser discharging into bag	Taste	4 sec	
KN95		Taste	about 7 sec	
Chin Gap N95		Taste	42 sec	The bag gave the mist more access to the chin area

Data Supplement - Photographs

Introduction

The following pages include photographs of the supplies, devices, enclosures, and testing procedures used. Each photograph includes a label describe the picture's content. Information from the paper may be necessary to fully understand the photos provided.

The photographs included are broken into categories:

Masks & Calibration Nebulizer
Enclosures
Other Supplies
Testing Photographs
Mask Fit

Masks & Calibration Nebulizer

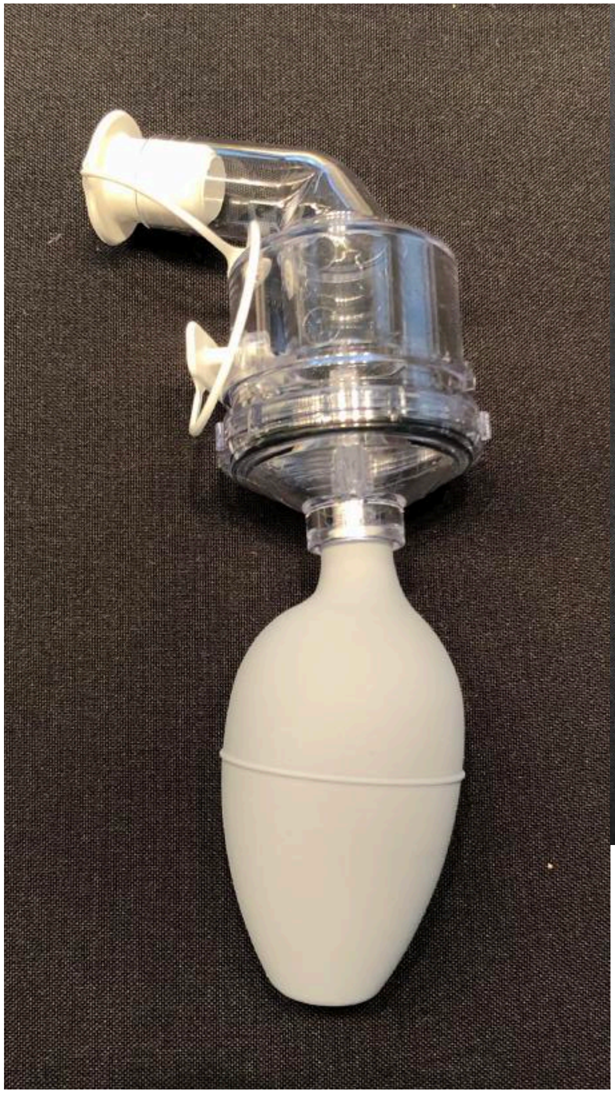

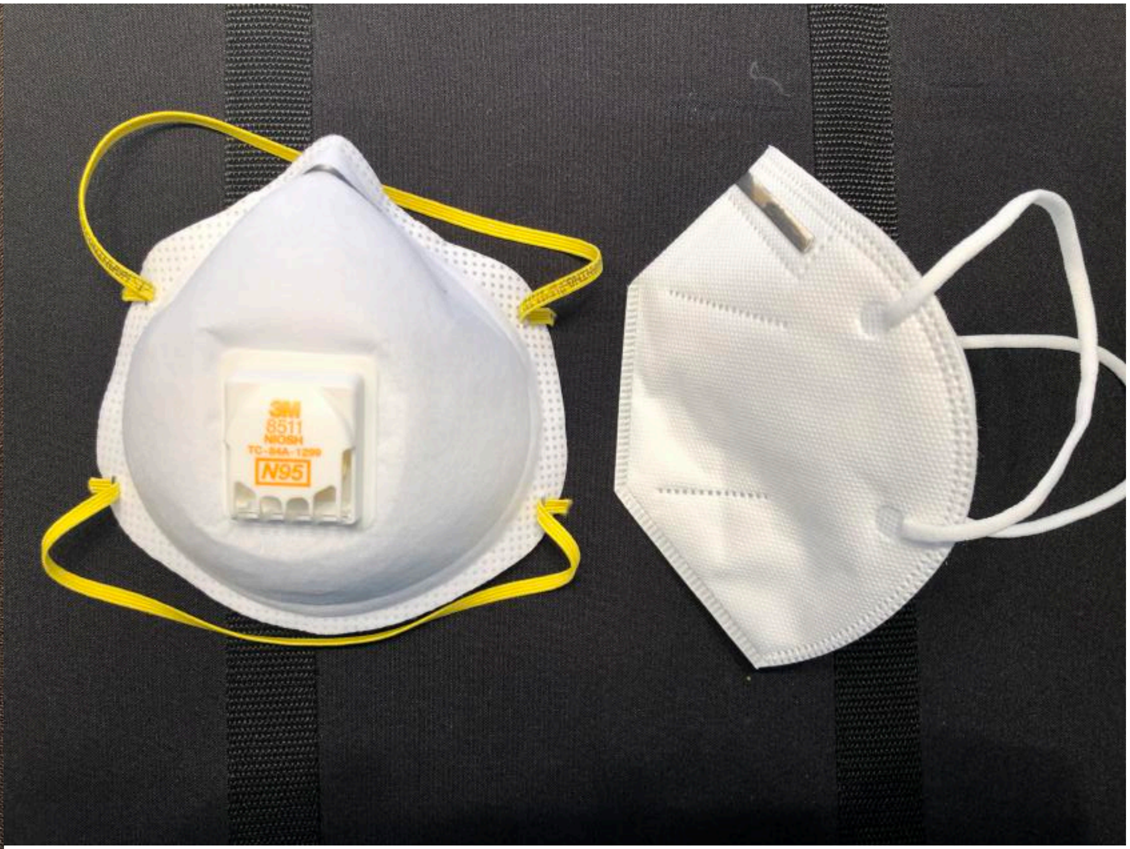

(left) A bulb nebulizer from a qualitative fit kit was used to ensure participants were able to taste the solutions and thus provide a control.

(above) The two masks participant wore, a N95 mask and KN95 mask. The KN95 mask fit extremely poorly.

Enclosures

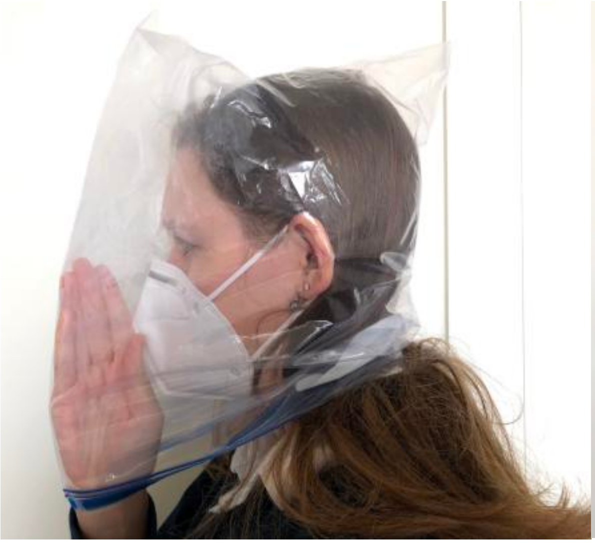

When using a plastic bag, ensure there is enough space between the end of the mask and the edge of the bag. Hold bag open with hand or testing device two help keep side of bag away from mask.

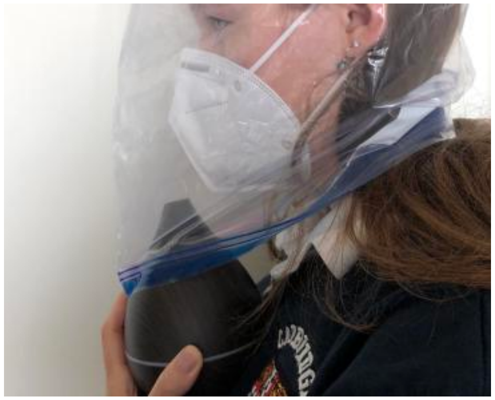

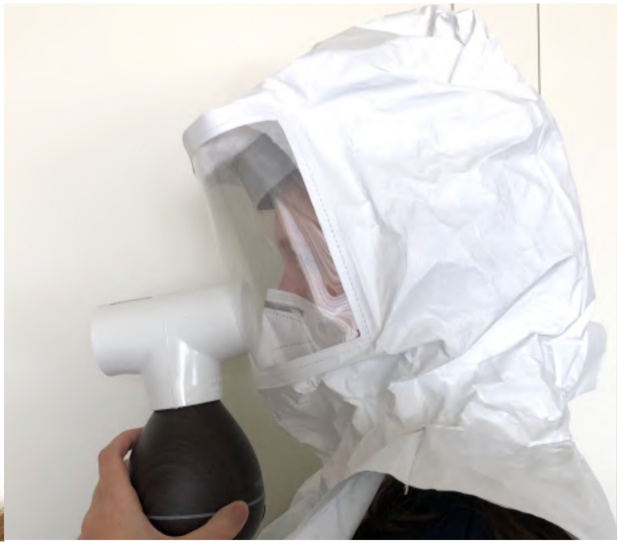

Qualitative testing hood includes hole in the front of the plastic shield by which solution to be administered. The only available hood was smaller than ideal for the subjects. This likely contributed to the single incorrect result reported. A 3-way PVC pipe was used to direct vertical flow into the hood.

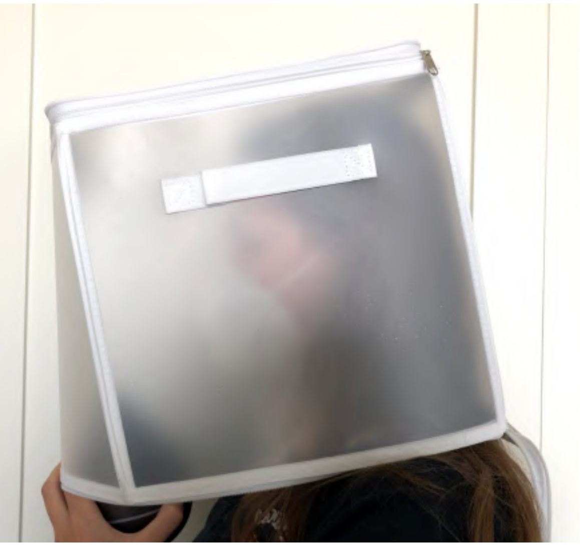

A box is over the head to create an enclosed space. This collapsable box had fabric seams which did not allow the mist to concentrate.

Other Supplies

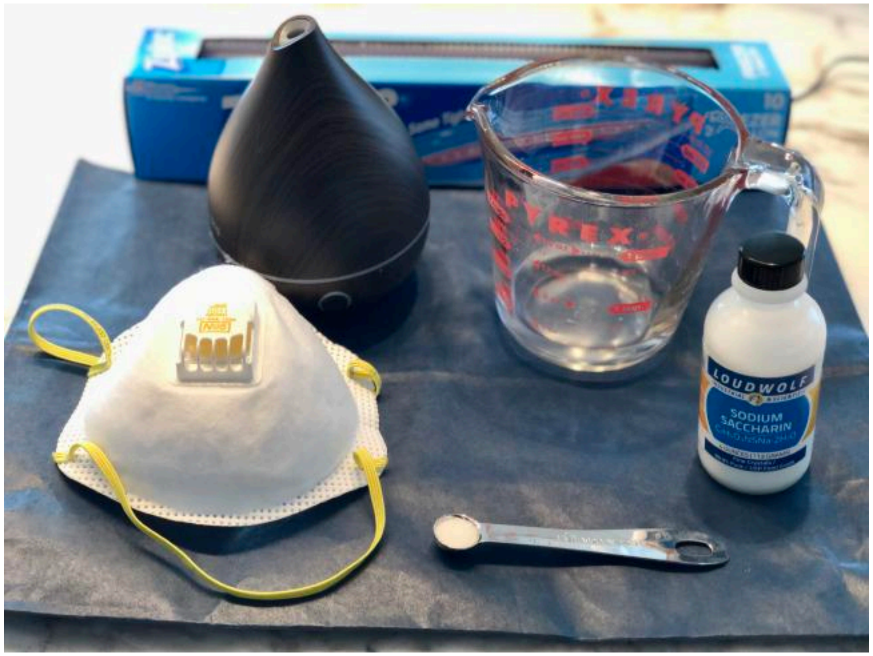

The supplies used for conducting a qualitative fit test with an aroma diffuser and sturdy bag shown above. Measuring cup filled with 100ml distilled water

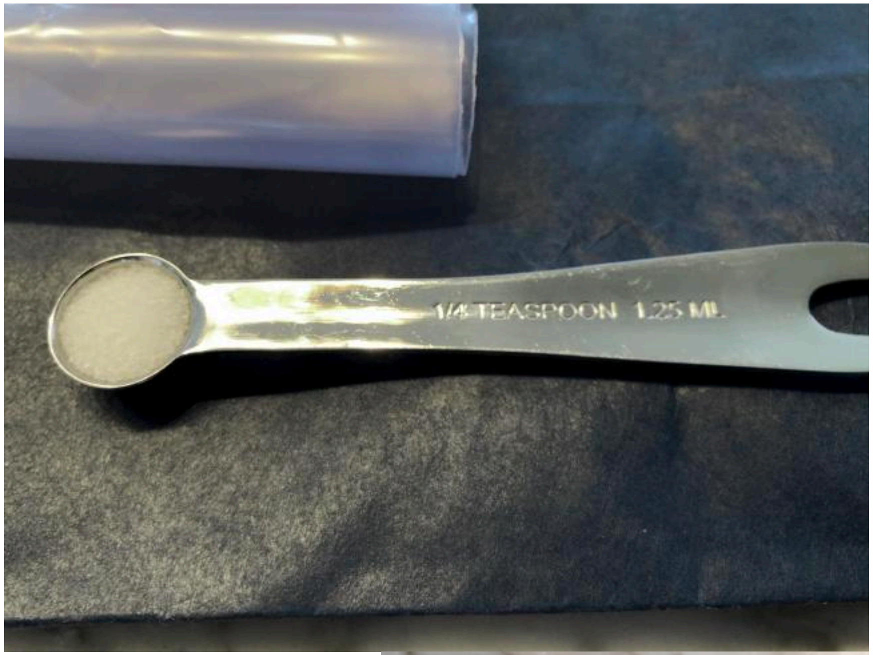

830mg of sodium saccharin measures just under 1/4 of a teaspoon. These photos show how 830mg of saccharin fills a standard 1/4 teaspoon.

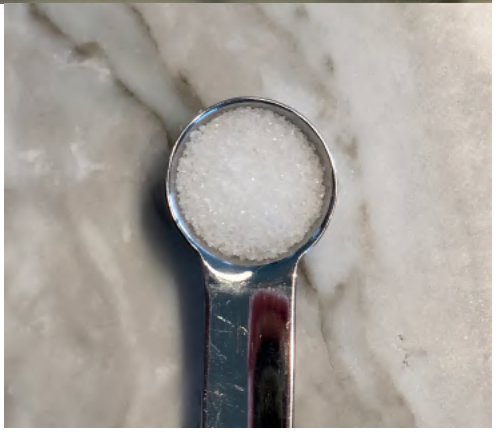

Testing Photographs

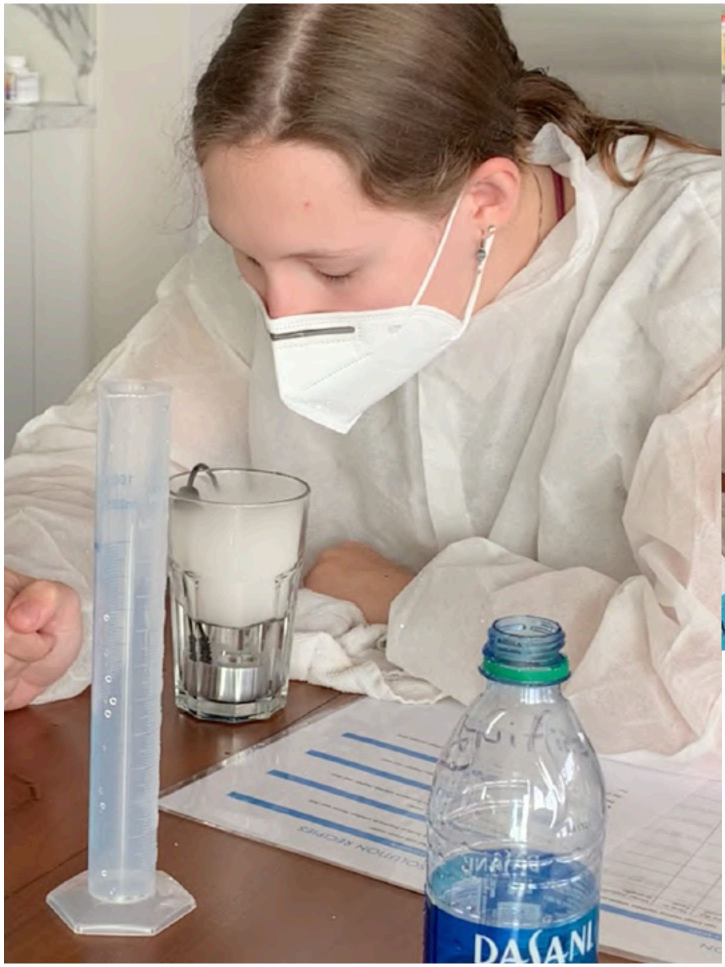

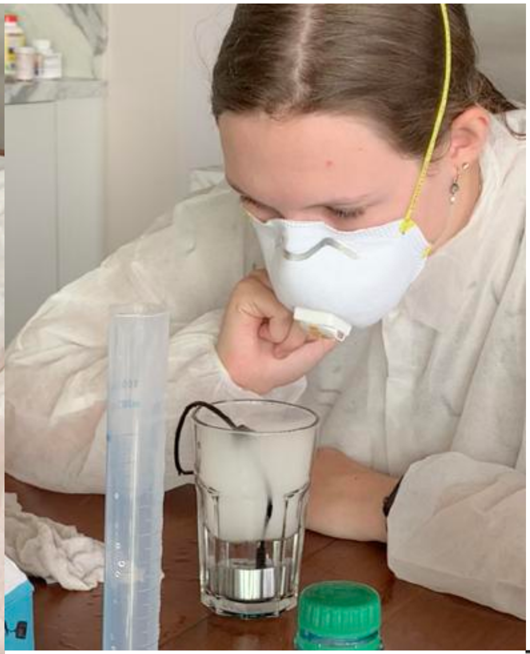

(left) Testing KN95 and N95 masks w/o enclosure (open air) and mist maker.

(right) Testing spray bottle with N95 mask w/o enclosure

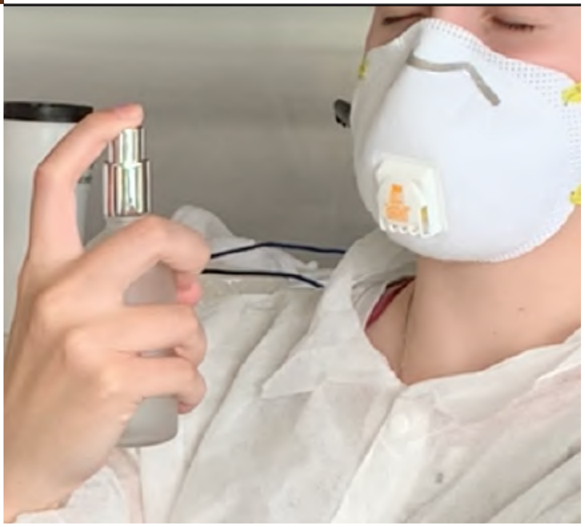

Mask Fit

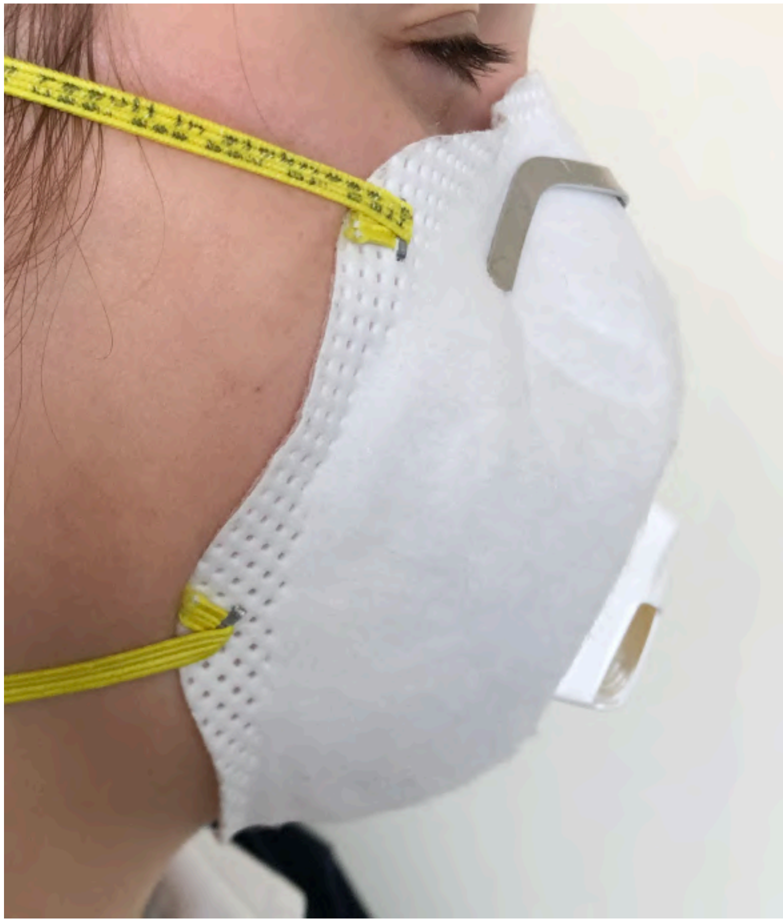

N95 mask used for testing had an excellent fit on participants 1 and 2.

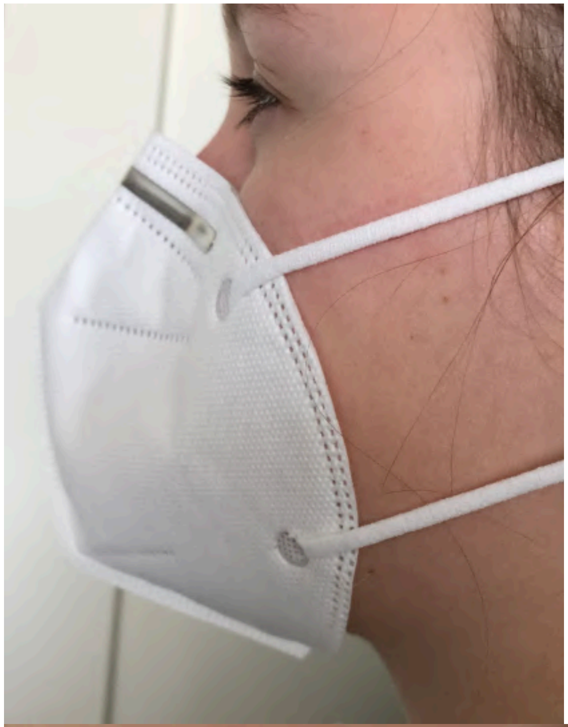

The KN95 mask, however, had a very poor fit on all participants. As you can see, the mask fits loosely and fails to confirm to the nose bridge.

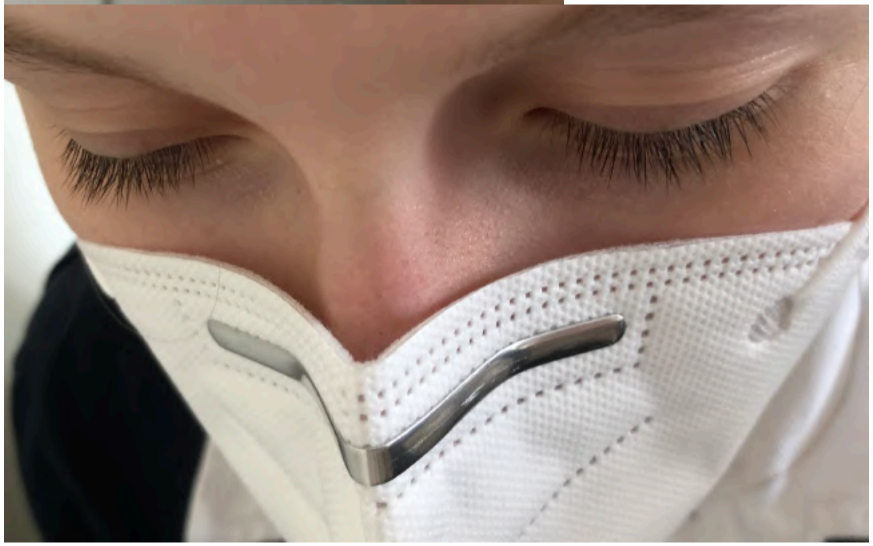

Data Supplement - Risk Analysis

These pages contain a preliminary FMEA risk analysis assessing the most successful devices and enclosures.

Device				
**Risk**	**Description**	**Probability**	**Severity**	**Actions to Minimize Risk**
Particles from aroma diffuser are larger than nebulizer	If the particles generated by the aroma diffuser are larger than the kit nebulizer, microscopic holes might not be detected. However, there is no good evidence that the traditional kit nebulizer provides the level of accuracy and precision needed to detect microscopic holes.	Moderate	Low	FUTURE WORK: Evaluate particle size from various aroma diffusers or obtain information from manufacturer's
Aroma diffuser builds up bacteria or other harmful substances	While the aroma diffusers are built to safely aerosolize particles, there is always the chance that the machine could become contaminated and aerosolize harmful pathogens or particles	Low	High	Clean aroma diffuser thoroughly before and after each use. Use aroma generator only for qualitative fit testing. Follow all manufacturer's guidelines on hygiene. Do not leave liquid standing in machine between uses. Clean and dry machine between uses.

Electric shocks from aroma diffuser if cord or electronic components get wet	The aroma diffuser is built to handle liquid. However, the cord and device juncture may cause issues if wet.	Low	Moderate	Follow all manufacturer's instructions. Keep testing solution away from electronic parts. Practice electronic safety precautions. Dry and parts of the device that become wet before plugging in or starting the device.
Different brands of aroma diffuser function differently	Different brands of aroma diffuser may function differently, say at different speeds, effecting the test	Low	Moderate	FUTURE WORK: Evaluate multiple brands for their effectiveness
Enclosure				
**Risk**	**Description**	**Probability**	**Severity**	**Actions to Minimize Risk**
Bag - Suffocation	If the particles generated by the aroma diffuser are larger than the kit nebulizer, very small microscopic gaps and holes might not be detected. However, there is no good evidence that the traditional kit nebulizer provides the level of accuracy and precision needed to detect these gaps.	Moderate	High	Plastic bags should only be used with great caution and by sound, able bodied adults who can safely and quickly remove the bag. The bottom of the bag should never be sealed. A second person should observe the testing and be able to assist in case of difficulty. Bags should always be rigid to help prevent suffocation.

Open Space - Lack of particle concentration	An open space may prevent the mist from becoming concentrated around the whole of the mask, thus failing to detect gaps	Medium	Medium	If no enclosure is used, aroma generator should be moved around mask and face area to ensure all sides of the mask are exposed to the mist
Bag - Lack of fit	Bag may not properly allow all of mask to be in mist for all users	Medium	Medium	A different bag or enclosure should be used.
Hood - Lack of fit	Hood may not allow all of mask to be in mist area for all users	Medium	Medium	A different enclosure should be used
Box - Does not concentrate particles	Box may not allow particles concentration to build up	Medium	Medium	Use a small box with airtight seams. Use a box with different dimensions.
